# Identifying the needs and optimal support for healthcare professionals after in-hospital cardiac arrest: a qualitative study

**DOI:** 10.3389/fmed.2026.1940522

**Published:** 2026-10-21

**Authors:** Sacha L. K. Roest, Kristian Krogh, Bo Løfgren, Kasper G. Lauridsen

**Affiliations:** 1 Research Center for Emergency Medicine, Department of Clinical Medicine, Aarhus University, Aarhus, Denmark; 2 Department of General Surgery, Viborg Regional Hospital, Viborg, Denmark; 3 Department of Anesthesiology and Intensive Care, Aarhus University Hospital, Aarhus, Denmark; 4 Department of Medicine, Randers Regional Hospital, Randers, Denmark; 5 Department of Anesthesiology and Critical Care Medicine, Children’s Hospital of Philadelphia, Philadelphia, PA, United States

**Keywords:** coping strategies, emotional impact, healthcare professionals, in-hospital cardiac arrest, qualitative study, resuscitation

## Abstract

**Introduction:**

The International Liaison Committee on Resuscitation has prioritized the well-being of healthcare professionals as a key step toward improving in-hospital cardiac arrest (IHCA) outcomes. However, evidence on how to ensure well-being is lacking. We aimed to investigate how IHCA affects healthcare professionals and to identify helpful coping strategies following IHCA.

**Methods:**

In this exploratory qualitative study, semi-structured individual interviews were conducted with nurses, doctors, and orderlies purposively recruited from two hospitals in the Central Denmark Region. Interviews were transcribed verbatim and analyzed using reflexive thematic analysis.

**Results:**

12 interviews were conducted. Data analysis revealed 6 main themes and 14 subthemes, grouped into 2 overarching themes: factors influencing the emotional impact of IHCA and coping strategies associated with IHCA. The emotional impact was defined by patient-related impact, reflecting the distress of treating young patients and children; contextual impact, such as equipment failures and unclear resuscitation decisions; and personal impact, reflecting the burden of a personal connection to the patient. Coping strategies included passive coping, comprising internal cognitive and emotional processes such as rationalizing outcomes; active coping, comprising deliberate actions such as post-event reflection and training; and reflective coping, comprising awareness of how interpersonal and organizational conditions influenced coping.

**Conclusion:**

This study indicates that healthcare professionals are emotionally affected by patient-related, contextual, and personal factors when dealing with IHCA, and they employ passive, active, and reflective coping strategies to navigate these situations. These findings may help institutions develop supportive systems to promote staff well-being after IHCA.

## 1 Introduction

In-hospital cardiac arrest (IHCA) is a high-stakes, time-critical event that may have profound consequences for both patients and healthcare professionals (1). In Denmark, more than 2,000 IHCAs occur annually, with a 30-day survival of approximately 28% (2, 3). As IHCAs occur across multiple departments, most healthcare professionals working in these settings may encounter an IHCA at some point in their career (2, 4, 5).

Experiencing IHCA may be a profoundly traumatic event, even for healthcare professionals (6–8). Several studies have shown that IHCAs impact the psychological well-being of healthcare professionals (8–10). Research exploring healthcare professionals’ experiences of resuscitation has further demonstrated that these events can be emotionally demanding and involve complex experiences related to the clinical situation, team interactions, patient outcomes, and the demands of resuscitation (11–13). However, evidence on the coping mechanisms and strategies used by healthcare professionals following IHCA is limited, and existing interventions lack robust empirical support, emphasizing the need for further research (14). Coping refers to the ways in which individuals manage and respond to stressful or emotionally demanding experiences. Previous research on resuscitation has identified a range of coping strategies used by healthcare professionals, including reflection, debriefing, and seeking social support (15). More broadly, research among acute and critical care nurses has identified a variety of coping strategies used to manage workplace stressors, while also demonstrating variation in how healthcare professionals cope with demanding clinical experiences (16). However, limited research has examined how healthcare professionals experience and perceive different coping strategies specifically following IHCA, including which strategies they find helpful in managing the emotional impact of these events. Understanding how IHCA affects healthcare professionals and the strategies they use to cope may provide valuable insights to help identify interventions and support mechanisms that promote resilience and reduce stress, thus improving well-being and sustainable healthcare practice (8–10). Given that the emotional impact of IHCA may contribute to burnout, stress, and work-related anxiety, it is crucial to identify methods that genuinely support staff in coping with the emotional stress associated with IHCA (8, 17, 18).

Increasing patient loads and inadequate staffing constitute a widespread international challenge (18, 19). Thus, the psychological well-being of healthcare professionals is recognized as a critical determinant for maintaining the longterm sustainability of healthcare systems (14, 18). Accordingly, the International Liaison Committee on Resuscitation (ILCOR) recently identified “ensure the well-being of health care professionals” as one of the ten prioritized steps to improve IHCA outcomes and quality of care (14). However, there is limited knowledge on how to ensure the well-being of healthcare professionals following IHCAs (9, 10, 14).

Accordingly, this study aimed to investigate how IHCA impacts healthcare professionals and to identify helpful strategies for coping following IHCA.

## 2 Methods

This study was designed as exploratory qualitative research, using individual semi-structured interviews. In accordance with Danish law, no ethical committee approval was required. Participants provided written consent (20). We followed the EQUATOR network standards for reporting qualitative research (21).

We included healthcare professionals from two hospitals in the Central Denmark Region—a university hospital functioning as a cardiac arrest center and a regional hospital, chosen to capture variation in size and context. Both hospitals have designated cardiac arrest teams. The sample size was determined based on the concept of information power (22). In this study, the aim was well-defined, and participants were purposively selected for their relevance to the research question. The sample size and the composition of the sample were therefore determined *a priori* rather than by an emergent stopping rule. Following data collection, the research team assessed the resulting dataset as providing sufficient information power to address the research question (22, 23). Participants were purposively sampled and invited by the research team via departmental email and personal contact. Recruitment was conducted using the same approach at both hospitals. The distribution of participants across professional groups was planned in advance, with 2 doctors, 2 nurses, and 2 orderlies recruited from each hospital to ensure balanced representation of professions involved in the cardiac arrest response. Orderlies, hospital workers responsible for e.g., patient transport, practical tasks, and performing chest compressions on cardiac arrest teams, were included because they are often overlooked in discussions of burnout and mental well-being (24, 25). We included ward nurses due to their close relationships with patients, who are usually the first to identify patients in cardiac arrest and initiate basic life support. We included physicians who either worked on the cardiac arrest team, or had experience caring for patients on wards where cardiac arrests occurred (24, 25). Participants were required to have been actively involved in an IHCA within the period ranging from 2 weeks to 18 months prior to the interviews. The timeframe was selected to balance participants’ opportunity for reflection and processing following an IHCA, yet considering that experiences with coping may alter several years after an event.

### 2.1 Data collection

Data were collected through semi-structured interviews conducted by the lead investigator. The lead investigator did not have a prior relationship with any of the participants. An interview guide was prepared before data collection and is provided in Supplementary File 1. The questions of the interview guide were made to explore how and why IHCAs emotionally impacted participants, as well as the coping mechanisms they utilized to navigate the associated feelings and thoughts. These questions were primarily open-ended, encouraging participants to express themselves freely, elaborate, and share their thoughts openly. The interview guide was developed based on the research question and the clinical and research experience of the research team and was not informed by a formal literature review. The interview guide was tested in a pilot interview, and edited to ensure the guide was easy to use and that the questions were clear and understandable for participants. The pilot interview was not included in the analysis. All interviews were conducted face-to-face in a private room at the respective hospital, with measures taken to minimize interruptions, and lasted approximately 45–60 min. All participants recounted specific IHCA events in detail. The interviews were audio recorded and transcribed verbatim by the lead investigator using TurboScribe.ai, which uses artificial intelligence (AI) and ensures secure data storage through website encryption (26). The interviews were conducted in Danish. All quotations presented in the Results and in the tables were translated from Danish into English by the lead investigator with the aim of keeping the meaning of the quotes. No field notes were taken.

Following AI-assisted transcription, the lead investigator reviewed each transcript against the original audio recordings and corrected minor errors to ensure an accurate verbatim record. The research team reviewed the first two interviews to determine whether any modifications were needed for the forthcoming interviews. Audio recordings were captured using the iPhone Voice Memos app® (Apple, Cupertino, United States), ensuring that recordings were encrypted during transit and stored in an encrypted format (27).

As the interviews could potentially reopen past traumas for the participants, they were encouraged to contact their immediate supervisor or the lead investigator to process their experiences if needed.

### 2.2 Data analysis

Data were analyzed using reflexive thematic analysis, following the six-phase methodology outlined by Braun and Clarke (23). This approach was chosen for its flexibility and focus on capturing participants’ lived experiences and meanings. The analysis was inductive, meaning that codes and themes were developed from the data rather than from a pre-existing framework.

The lead investigator, a sixth-year medical student with several years of experience as a certified basic life support instructor and locum physician in psychiatry, conducted all interviews and performed the initial coding. The lead investigator first familiarized herself with each transcript and generated initial codes freely from the data, with the research question guiding the relevance of the codes and without predefined codes or categories. Reflexivity was maintained throughout by considering how the research team's clinical backgrounds and experiences with in-hospital cardiac arrest (IHCA) might influence interpretation. These considerations were discussed within the research team during the analysis, allowing assumptions and potential biases to be challenged and alternative perspectives to be considered. In particular, the lead investigator's experience as a certified basic life support instructor may have sensitized the analysis to training and skill acquisition as coping resources, while the remaining authors’ backgrounds in resuscitation research, education, and debriefing may have oriented interpretation toward organizational and team-level explanations. These potential influences were made explicit and revisited when codes and themes were reviewed. Codes and emerging themes were subsequently discussed within the research team. The other research team members were all experienced clinician-scientists and educators with extensive experience in resuscitation and qualitative research methodology. The research team contributed by challenging assumptions, incorporating diverse perspectives, and strengthening interpretation.

All transcripts were coded systematically using NVivo 14 (28). A primarily semantic approach was chosen to remain closely grounded in participants’ explicit accounts of their experiences and coping following IHCA, while latent coding was used when underlying meanings were relevant to the research question (23). Codes were documented in a codebook maintained by the lead investigator to document and organize the coding process. After initial coding, codes were grouped into broader patterns, and preliminary themes were developed. Initial coding generated 82 codes across the 12 transcripts, which were clustered into candidate subthemes on the basis of shared meaning. The coding and theme development process was iterative, with the different phases of analysis often revisited as the analysis progressed. These themes were then reviewed, discussed, and refined collaboratively with the research team to ensure depth, coherence, and credibility.

## 3 Results

Between March 27 and April 24, 2024, we conducted interviews with 12 healthcare professionals—4 doctors, 4 nurses, and 4 orderlies. All invited participants agreed to take part. Purposeful sampling ensured diversity across departments, genders, and experience levels. Table 1 presents the participants’ baseline demographics. Data analysis revealed 6 main themes and 14 subthemes, grouped into 2 overarching themes: factors contributing to the emotional impact of IHCA and coping strategies associated with IHCA.

**Table 1:** Baseline demographics of the participants. Clinical experience is presented as the median with Q1 and Q3 representing the first and third quartiles. All other demographic data are reported as *n* (%).

All healthcare professionals *n* = 12	
Clinical experience [median (Q1;Q3)] years	7.25 (4.75; 20)
Gender, female	7 (58.3%)
Location, Aarhus University Hospital	6 (50%)
Location, Randers Regional Hospital	6 (50%)
Profession, doctors	4 (33.3%)
Profession, nurses	4 (33.3%)
Profession, orderlies	4 (33.3%)

### 3.1 Emotional impact of IHCA

Overall, 3 themes and 7 subthemes (Table 2) were identified relating to factors contributing to the emotional impact of IHCAs. The themes were patient-related impact, contextual impact, and personal impact.

**Table 2:** Themes and subthemes concerning the most significant factors affecting healthcare professionals in managing in-hospital cardiac arrests.

Theme	Subthemes	Description	Supporting quote
Patient-related impact	Age	Young patients with IHCA affect healthcare professionals more deeply.	*Orderly 3: “Yes, if children are involved, it can affect me more deeply than other things.”*
	Next of kin	Dealing with relatives of deceased patients affects healthcare professionals more deeply.	*Doctor 3: “And having to call the wife, hearing the children crying in the background, knowing their father is dead. There were many aspects to it. It still affects me today.“*
Contextual impact	Equipment	Equipment malfunction during IHCA affects healthcare professionals more deeply.	*Doctor 1: “Yes, when we had to deliver the first shock, for some reason, the pads weren't connected to the defibrillator.” … “And you know, it's just one of those things… I mean, it's something that normally doesn't happen.”*
	Team	When there are disruptions in the IHCA team, healthcare professionals are more affected.	*Nurse 4: “But I think our biggest frustration was that we didn't have a doctor. You know, because they come from anesthesia, the cardiac arrest team that arrives. And they're like, “who is the patient?”.”*
	Location	Healthcare professionals experience emotional distress when the IHCA environment is unpredictable.	*Nurse 2: “It was someone who had collapsed in the bathroom. There wasn't much space. We had to get them into bed, and it was just chaos, and there were many people.”*
Personal impact	First IHCA	The initial IHCA experience significantly affects healthcare professionals emotionally.	*Orderly 1: “Yes, my first cardiac arrest call, but I think it's also because I remember it as I do. And I don't think I could ever let go of it.”*
	Patient relation	IHCA emotionally impacts healthcare professionals more when there is a personal connection with the patient or they remind them of loved ones.	*Doctor 1: “ You won’t die on my shift. Because it was him. So, in that way, it obviously affected me too. I think we were all like… It was someone we knew well, and who I think everyone liked. That certainly means something.”*

IHCA, In-hospital cardiac arrest.

#### 3.1.1 Patient-related impact (age, next of kin)

Ten participants reported that treating young patients or children with IHCA had the greatest emotional impact. Three participants had personally experienced a pediatric IHCA, while four participants who had not encountered such cases described dreading these experiences. A few participants reported avoiding work in departments where pediatric IHCAs were more likely to occur.

Doctor 2: “The scream when she realized that her child had died. You never forget that. It's probably one of the most horrible things I have experienced. I mean, aside from when your own loved ones die and such. But it's on that level.”

#### 3.1.2 Contextual impact (equipment, team, location)

Contextual factors also significantly influenced the emotional impact of IHCAs. All participants reported heightened emotional distress when the IHCA context deviated from the norm. This included equipment malfunctions, unusual locations, or suboptimal team performance. Nurses, in particular, highlighted frustration related to unclear ‘do not attempt cardiopulmonary resuscitation’ decisions.

Nurse 4: “It affected some of my colleagues more deeply; they found it very frustrating that no decision had been made about resuscitation. I also found it really frustrating.”

#### 3.1.3 Personal impact (first IHCA, patient relations)

All participants experienced a significant emotional impact when they had a personal connection to the patient. This connection could arise from knowing the patient personally, the patient having had an extended hospital stay, or the healthcare professional being able to relate to the patient on a personal level. Participants also described their first experience with an IHCA as particularly emotionally impactful.

Orderly 3: “When you're involved with a patient for several hours, and then the patient suddenly goes into cardiac arrest, that's what really sticks with me. Those are the ones I remember.”

### 3.2 Coping strategies associated with IHCA

Overall, 3 primary themes and 7 subthemes (Table 3) were identified, representing different ways in which healthcare professionals described coping with the emotional impact of IHCA. The themes were distinguished according to how participants described their coping: passive coping involved internal cognitive and emotional processes without deliberate action; active coping involved deliberate actions taken to manage or process the experience; and reflective coping involved awareness of and reflection on how interpersonal and organizational conditions influenced their ability to cope.

**Table 3:** Themes and subthemes concerning the coping mechanisms employed by healthcare professionals in managing in-hospital cardiac arrest.

Themes	Subthemes	Description	Supporting quote
Passive coping	Justification	Healthcare professionals found it easier to cope by rationalizing both the patient outcome and the contextual factors of the IHCA.	*Doctor 4: “Well, I think I try to justify things. So that's one of my mechanisms.”*
	Distancing	Healthcare professionals employed emotional distancing to detach from the emotional aspects of IHCA.	*Doctor 1: “Then the shutters go down. And then I do everything I can for them, as a professional, but without emotionally involving myself at all. Because then you couldn*’*t handle it.”*
	Exposure	Healthcare professionals described repeated exposure to IHCAs as making subsequent events easier to handle and less emotionally challenging.	*Doctor 1: “I actually think that as you’ve been in these situations many times, it*’*s not something that, so to speak, emotionally affects you.”*
Active coping	Post-event reflection	Healthcare professionals use post-event reflection to cope with emotional stress of IHCA, varying in depth and intensity.	*Nurse 3: “I just think I’m the type where it helps to talk about it. Whether they can answer something professionally or just, like I do with the relatives, just be there and accommodate their feelings”*
	Addressing educational needs	Healthcare professionals cope by pursuing additional training and education, and refining resuscitation skills.	*Doctor 3: “I think scenario training in general is really good because you get a bit nervous; you feel it on your own body somehow, even though it's a doll or an actor lying there.”*
Reflective coping	Work environment	Healthcare professionals cope better with IHCA in a positive work environment with competent, supportive colleagues.	*Orderly 1: “So, the fact that the cardiac arrest team comes together and tries to bond, to meet each other at eye level and connect with one another by asking, ‘What*’*s your name?’ I can see that it makes a huge difference. By now, I also know some of the anesthesiology team because I’ve been out with them a few times. And simply knowing them, for example knowing their first name, makes a world of difference.”*
	Emotional awareness	Healthcare professionals reflected on the importance of a work environment with competent, supportive colleagues and psychological safety for their ability to cope with IHCA.	*Doctor 2: “If they’re not people I trust, they shouldn*’*t have ownership of what I feel. So, I think it*’*s something that really depends on who you are as a person, but you just need to know that you should only do it when you’re ready for it. No one should force you to do anything.”*

IHCA, In-hospital cardiac arrest.

#### 3.2.1 Passive coping (justification, distancing, exposure)

Passive coping was characterized by internal cognitive and emotional processes that helped healthcare professionals manage the emotional impact of IHCA without deliberate action. Subthemes included justification, where they rationalized the cardiac arrest and unsuccessful resuscitation based on patient and contextual factors, such as comorbidities or the relief of suffering; distancing, where they emotionally detached from the event and separated their personal and professional lives; and exposure, where healthcare professionals described repeated exposure to IHCAs as making subsequent events easier to handle. Although exposure was not described as a deliberate coping action, participants experienced repeated exposure as a process that made subsequent IHCAs less emotionally challenging.

All participants emphasized the importance of considering both the outcome and contextual factors when coping.

Doctor 2: “So it's like if I've been in a cardiac arrest situation, I reassure myself by thinking that I did the right thing, or I did this or that. So I think that's my mechanism. I go back and look at whether things were under control and what actually happened.”

Another aspect of coping mechanisms was emotional distancing. Eleven healthcare professionals employed this strategy to detach themselves emotionally from IHCAs.

Orderly 1: “Now I am not myself. Now I am the one performing chest compressions.”

#### 3.2.2 Active coping (post-event reflection, addressing educational needs)

Active coping involved deliberate actions taken by healthcare professionals to manage or process the emotional impact of IHCA. Subthemes included post-event reflection, where participants actively processed specific IHCA experiences by discussing the incident with peers, and addressing educational needs, in which participants pursued additional training and refined resuscitation skills.

Reflection sometimes occurred through systematic department-led debriefings. Eight participants found it helpful to discuss their emotional experiences with peers, while five participants found discussing the IHCA scenario step by step helpful in processing the event. Two participants reported that they did not derive benefit from discussing their emotions during these debriefings. Six participants believed that systematizing coping mechanisms could improve coping skills. Often, reflection was initiated independently, preferably with a colleague from the same profession who had also participated in the IHCA. All 12 participants found it easier to process the emotional impact when sharing experiences with co-workers who had experienced similar events.

Orderly 3: “Well, what works best for me is clearly that if something affects me, it’s talking to my colleague who is in the same profession as me.”

No departments had a standardized debriefing protocol. Occasionally, multiprofessional cold debriefings were organized, but orderlies reported these as less effective for emotional coping, feeling unsafe or unable to express their emotions.

Orderly 1: “You are also standing with people who are a bit more educated than yourself. So, even if you might want to focus on the sensitive stuff, you can't. Because you don't really feel that it interests them.”

#### 3.2.3 Reflective coping (work environment, emotional awareness)

Reflective coping was characterized by awareness of and reflection on how interpersonal and organizational conditions influenced healthcare professionals’ ability to cope with IHCA. Subthemes included work environment, which captured participants’ reflections on the importance of competent and trustworthy colleagues and psychological safety for coping, and emotional awareness, which concerned recognizing and understanding their own and others’ emotional responses to IHCA.

Doctor 2: “I believe that one should greatly respect that people need to be ready for it. And one must not exploit people who are emotionally fragile. One should allow them to refrain from talking about it if they wish, and then they can discuss it with someone they trust.”

Younger doctors reported that morning conferences were an unsafe environment, expressing discomfort in sharing experiences as IHCA team leaders during night shifts in front of many colleagues. Participants emphasized that trusting relationships, psychological safety, and competent colleagues are crucial for coping, providing confidence and reassurance during IHCA events.

Orderly 2: “Well, when I thought about it, my colleagues were actually really good at asking how I was doing after the first episodes with cardiac arrest and such. But again, and this is a recurring theme, it is just really important to have good colleagues.”

## 4 Discussion

Our study offers valuable qualitative insights by identifying 6 main themes and 14 subthemes that illustrate both the emotional effects and the coping strategies employed by healthcare professionals.

Overall, the emotional impact of IHCAs on healthcare professionals remains underexplored (14, 29). In our study, patient-related, contextual, and personal factors were identified as important contributors to the emotional impact of IHCA. Participants particularly emphasized the extreme impact when the patient was a child. This reaction may reflect broader cultural and social perceptions of pediatric deaths as especially tragic and unexpected. Likewise, previous studies have demonstrated that nurses involved in IHCAs experience heightened stress when resuscitating a child or younger patient, during equipment failures, and during the early stages of their careers (7, 30). The emotional impact on healthcare professionals in our study was notable. A few participants explicitly expressed reluctance to work in departments where IHCAs involving children or younger patients are more likely to occur. Such avoidance may reflect apprehension associated with the possibility of encountering a particularly challenging IHCA. Although PTSD, burnout, compassion fatigue, and other stress-related conditions are distinct constructs, they may manifest as emotional or psychological distress (8, 17, 18). Our participants described such distress in relation to IHCA. However, we are unable to attribute these experiences to any specific condition in our data. Previous research has found PTSD symptoms in 9.6% of emergency care staff after attending an IHCA, with junior staff being particularly vulnerable (8). Additionally, one study identified a link between post-IHCA stress and compassion fatigue, suggesting that the stress surrounding IHCAs may contribute to emotional exhaustion among critical care nurses (17). A meta-analysis by Hodkinson et al. further underscores this concern, showing that physician burnout is associated with diminished organizational function and increased turnover, ultimately impacting patient care (18). Together, existing literature and insights from this study highlight the importance of acknowledging the emotional challenges associated with IHCA and emphasizing institutional strategies to support staff well-being.

Our findings extend previous research on healthcare professionals’ experiences of resuscitation by providing further insight into how healthcare professionals cope with IHCA. Previous research has identified a range of coping strategies, including reflection, seeking social support, and debriefing (11, 15), while our findings further highlight how coping is influenced by interpersonal and organizational conditions. Three aspects of these findings appear particularly novel. First, orderlies are rarely represented in studies of the well-being of IHCA responders, and our findings indicate that their experience of multidisciplinary debriefing differs from that of doctors and nurses. Second, our findings distinguish between support being offered and support being usable, as a debriefing that is available may still be inaccessible to those who do not experience the setting as psychologically safe. Third, junior doctors described the morning conference, a routine forum for case review, as an unsafe setting in which to discuss arrests they had led. We found that healthcare professionals coping with IHCA often engaged in passive distancing or rationalized both the outcome and the surrounding circumstances. Such responses may serve as secondary defense mechanisms. Psychological theorists have suggested that avoidant coping strategies — such as ignoring distressing situations or suppressing emotions — can increase the risk of secondary traumatization (31). Furthermore, some healthcare professionals had not considered coping mechanisms when dealing with IHCA. Yet, they found it helpful to discuss the emotional impact of IHCA, while some became emotionally distressed during the interviews. Such emotional responses may reflect the difficulty of recalling challenging resuscitations or patient deaths and do not necessarily indicate inadequate coping. In contrast, studies have found that active, task-oriented coping strategies (e.g., training) are associated with a decreased risk of burnout (32), while social support has been identified as an important coping strategy following resuscitation (15).

All healthcare professionals used post-event reflection as a coping strategy, with team debriefings and peer discussions being highly valued. All participants felt able to seek support from colleagues after an IHCA, and some participants emphasized that coping should not be their sole responsibility and called for a more proactive role from hospitals. Importantly, those with established support networks may be better equipped to seek help, but newer staff or those without such resources may struggle to access appropriate support. Participants generally indicated a lack of regular debriefings following IHCA and appreciated when debriefings were held. However, our findings also highlight the complexity of interdisciplinary debriefings, as some orderlies reported difficulties sharing their thoughts and feelings with other professionals with different educational and professional backgrounds, which may reflect perceived hierarchies within the medical team.

Our study also highlighted the significant influence of institutional and cultural factors on coping. A supportive work environment with a culture of emotional openness facilitated greater utilization of post-reflection, resulting in reduced apprehension. This aligns with previous findings suggesting that collaborative interventions involving management and peers within the service are essential for stress reduction in emergency care (33). These findings underscore the need to address and systematize the institutional foundation for effective coping strategies for IHCAs, ensuring the well-being of healthcare professionals. In this regard, some participants suggested that repeated exposure to IHCAs may contribute to emotional strain among more experienced colleagues (32). Although all healthcare professionals found IHCAs easier to handle with increasing experience, repeated exposure may still contribute to emotional strain over time. Thus, becoming more experienced may facilitate coping with individual IHCAs without preventing cumulative emotional strain. This reflects an awareness that stress can accumulate over time and adversely affect mental well-being (31), underscoring the importance of acknowledging and supporting the emotional needs of healthcare professionals at various career stages.

### 4.1 Considerations to ensure the well-being of healthcare professionals

This study provides qualitative insights into ILCOR's prioritized step of ensuring the well-being of healthcare professionals (14). Based on the participant reflections, we have provided suggestions to hospitals on how to ensure the well-being of their employees, of which no evidence was identified by ILCOR (Table 4). Our findings suggest that interventions should not focus solely on individual coping, but should also consider the interpersonal and organizational conditions that influence how healthcare professionals experience and use available support. Although performance debriefings may be associated with improved survival outcomes after cardiac arrest (34, 35), performance debriefing and emotional support serve different purposes and may require different approaches (31, 35). Debriefings following IHCA may be structured to focus on performance or psychological well-being and may be conducted as “hot debriefings” (i.e., immediately after the event) or cold debriefings (several days after the event).

**Table 4:** Suggestions for supporting the well-being of healthcare professionals dealing with in-hospital cardiac arrest.

Suggestion	Description
Implement hot defusing sessions	Hold brief post-IHCA sessions focused specifically on emotional distress, distinct from performance-focused debriefing, to provide immediate support, with flexibility for individual support when needed.
Offer profession-specific and peer-group support options	Allow healthcare professionals to receive support with colleagues of their own profession where multidisciplinary settings are not experienced as psychologically safe.
Train debriefing facilitators in psychological safety	Ensure that those leading post-IHCA debriefings are prepared to manage professional hierarchies and to invite contributions from all professional groups.
Integrate emotionally challenging scenarios into IHCA training	Incorporate scenarios that simulate heightened emotional distress, helping staff practice coping strategies to identify what works best for them.
Launch a department-wide campaign on the emotional impact of IHCA	Encourage experienced colleagues to share their insights, fostering emotional awareness and open discussions.
Ensure staff familiarity with team, equipment, and environment	Strengthen preparedness by making sure employees feel confident in their surroundings and resources.
Recognize that experience does not eliminate the need for support	Acknowledge that even highly experienced professionals may need coping strategies after an IHCA.

Many participants expressed a desire for systematic team debriefing, not only to improve resuscitation skills but also to create an emotionally supportive environment for sharing feelings. Such debriefings may be organized as a “hot debriefing” or defusing immediately after the cardiac arrest to assess the well-being of the providers and identify if anyone requires further assistance which is now also recommended by the European Resuscitation Council (36). Additionally, such defusings may be used to identify cases where a more focused debriefing is organized within days or weeks after the cardiac arrest. Providing flexibility in how and with whom healthcare professionals receive support may be particularly important when multidisciplinary settings are not experienced as psychologically safe. Participants believed that regular team debriefings after every IHCA could enhance interprofessional relationships and help establish a routine, making it easier to manage the emotional impact of emotionally challenging IHCAs. Moreover, training appears to be an important target, as a lack of training in handling certain contextual factors may be associated with a more severe emotional impact. Training was also considered as a part of coping following an IHCA, which is supported by other findings that training is a favorable coping strategy in emergency care (32).

Creating a psychologically safe work environment is also crucial. This can be achieved by educating healthcare professionals on coping mechanisms and fostering an environment that normalizes and accepts emotional vulnerability. Support should also be flexible and accessible in ways that healthcare professionals feel comfortable with. Implementing a range of interventions, regardless of complexity, could significantly improve the well-being of healthcare professionals dealing with IHCA.

Further research is needed on effective strategies for coping with IHCA, including implementation, evaluation, and exploration of new approaches. This study paves the way for future investigations that will provide nuanced insights supporting healthcare professionals in managing IHCA effectively.

### 4.2 Limitations

As an exploratory qualitative study, the findings are shaped by participants’ accounts and the researchers’ interpretive engagement with the data. To strengthen credibility, analytic decisions, codes, and emerging themes were discussed within the research team throughout the analytic process. However, as interviews and initial coding were conducted by one researcher, early interpretations may have been influenced by that researcher's background.

Although the sample size was limited, the study was guided by the concept of information power, and the inclusion of participants with diverse professional backgrounds and experience levels allowed for a broad exploration of healthcare professionals’ perspectives. However, this study was not powered to support conclusions regarding differences in perspectives between professional groups, departments, or levels of experience.

The interval between IHCA involvement and interview ranged from 2 weeks to 18 months, which may have influenced recall and how participants interpreted their experiences. In addition, participants willing to discuss emotional responses after IHCA may have differed from those who did not participate, for example by being more reflective or more comfortable discussing distress. Finally, some participants experienced distress when recalling challenging events. To mitigate this, interviews were conducted with care, participants were encouraged to pause or adjust the conversation as needed, and post-interview support was offered.

## 5 Conclusion

This study indicates that healthcare professionals are emotionally affected by patient-related, contextual, and personal factors when dealing with IHCA. To cope, professionals draw on a range of strategies, including passive distancing, active approaches like training, and reflective practices such as peer discussions and team debriefings. These findings underscore the need for organizational support systems and further research to promote the well-being of healthcare providers.

## Data Availability

Deidentified raw data supporting the conclusions of this article may be made available by the authors upon reasonable request.
