# Supplementary material for: Identifying the needs and optimal support for healthcare professionals after in-hospital cardiac arrest: a qualitative study
Source: Front Med (Lausanne). 2026 Oct 21;13:1940522. doi: 10.3389/fmed.2026.1940522 (PMC13731201; doi:10.3389/fmed.2026.1940522)
Supplement: Supplementary file 1 [file Datasheet1.pdf]

INTERVIEWGUIDE	
(Obtain consent form and start audio recording)	
**Introduction**	
Presentation of the interviewer	*My name is Sacha and I am a medical student. I am doing my master's thesis this semester and will soon be finished as a medical doctor.*
Purpose	*The purpose of this interview is to learn about how you process the encounters and feelings in relation to in-hospital cardiac arrest events and what experience you have of the topic.*
Timeframe	*30-40 minutes have been allocated for the interview.*
Premises	*The interview will be audio recorded, transcribed, anonymized and then analyzed.*
Presentation of the interviewee	*Will you tell me a bit about yourself?*<br>*(profession + professional experience)*
**Research question**	
Experience	*' How many in-hospital cardiac arrests (IHCA) have you been a part of?*
IHCA events	*' When was the last time you were involved in an IHCA, and will you tell me about what happened?*<br>*' Try tell me about a IHCA that had great emotional impact on you?*<br>*' When did this event happen?*<br>*' How come this was the event you chose to tell me about?*<br>*' Which impression/thoughts/emotions does this episode evoke in you?*<br>*' What significance does this event hold for you today as you go to work?*<br>*' How does it affect your everyday life?*<br>*'What emotional difference is there between the most recent IHCA and the one we are talking about now?*
Processing	*'What was done at the hospital/your department to ensure you could talk about and process the incident?*<br>*' What do you do yourself to process the experiences and emotions after an IHCA?*<br>*' What kind of processing works well for you?*<br>*' Do you do anything consciously to process IHCA?*<br>*' Are there things you do unconsciously to process IHCA?*<br>*' What kind of processing does not work for you?*<br>*' How has your way of processing IHCA evolved throughout your professional life?*
Future initiatives	*' What initiatives for processing IHCA do you think would be helpful for you?*
Additional information	*' Is there anything we haven't talked about yet that you think we should discuss?*
**Conclusion**	
Closure	*Now we have reached the end of this interview.*
Ensuring emotional well-being	*What we have talked about today might cause emotional impact. It is important for me to ensure that you are okay. Do you have anyone at work you can talk to? Both I and my supervisors, who are doctors, are available to provide the support you might need. If any comments, thoughts, or questions arise after the interview, you are more than welcome to call or write to me.*
